# Identification and validation of DNA methylation markers to predict axillary lymph node metastasis of breast cancer

**DOI:** 10.1371/journal.pone.0278270

**Published:** 2022-12-01

**Authors:** Jianguo Luo, Shaojun Chen, Jingsen Chen, Yige Zhou, Fei He, Enli Wang

**Affiliations:** Department of Breast Surgery, Shenzhen Maternity & Child Healthcare Hospital of Southern Medical University, Shenzhen, Guangdong, China; American Society for Investigative Pathology, UNITED STATES

## Abstract

**Background:**

Axillary lymph node metastasis (ALNM) is one of the most important prognostic factors for breast cancer patients, and DNA methylation is involved in ALNM of breast cancer. However, the methylation profile of breast cancer ALNM remains unknown.

**Methods:**

Breast cancer tissues were collected from patients with and without ALNM. We investigated the genome-wide DNA methylation profile in breast cancer with and without ALNM using reduced representation bisulfite sequencing (RRBS). Then, differentially methylated regions (DMRs) were verified by targeted bisulfite sequencing.

**Results:**

A total of 21491 DMRs were identified between the lymph node positive group and negative group. Compared to the LN-negative breast cancer, LN-positive breast cancer had 10,920 hypermethylated DMRs and 10,571 hypomethylated DMRs. Then, 10 DMRs in the gene promoter region were detected by targeted bisulfite sequencing, these gene included HOXA5, PTOV1-AS1, RHOF, PAX6, GSTP1, RASGRF2, AKR1B1, BNIP3, CRMP1, ING5. Compared with negative lymph node, the promoter methylation levels of RASGRF2, AKR1B1 and CRMP1 increased in positive lymph node, while the promoter methylation level of RHOF decreased in positive lymph node. In addition, Cancer Genome Atlas (TCGA) data showed that RASGRF2, AKR1B1 and CRMP1 were low expressed in breast Cancer tissues, while RHOF was high expressed in breast Cancer tissues. Furthermore, in addition to highly methylated AKR1B1, RASGRF2 and CRMP1 gene promoters, BNIP3, GSTP1, HOXA5 and PAX6 gene promoters were also methylated in ER-positive and HER2-negative breast cancer with ALNM.

**Conclusions:**

When compared to negative lymph node breast cancer, the positive lymph node breast cancer has a differential methylation status. Promoter methylation of RASGRF2, AKR1B1, CRMP1 and RHOF in lymph node positive breast cancer tissues was significantly different from that in lymph node negative breast cancer tissues. AKR1B1, RASGRF2, CRMP1, BNIP3, GSTP1, HOXA5 and PAX6 genes were methylated in ER-positive and HER2-negative breast cancer with ALNM. The study provides an important biological base for understanding breast cancer with ALNM and developing therapeutic targets for breast cancer with ALNM.

## Introduction

Breast cancer is the most common malignancy in women worldwide, accounting for 25.1% of all cancers in women [1]. And the incidence of breast cancer is on the rise year by year, which affects women’s the health and life quality [2–4]. In recent years, although the treatment of breast cancer has been improved, the recurrence and metastasis rate of breast cancer is high, and the prognosis is still poor. Furthermore, the 5-year survival rate of patients with metastatic breast cancer remains less than 30% [5]. Therefore, early diagnosis is very important for its treatment and prognosis.

Metastasis is one of the characteristics of malignant tumors, which determines the course of treatment and the prognosis of cancer [6]. Metastasis of tumor tissue to other body organs accounts for the majorities of deaths in breast cancer patients, with axillary lymph node metastasis (ALNM) being the most common metastatic disease [7, 8]. In addition, lymph node metastasis (LNM) is one of the most important prognostic factors for breast cancer patients [9].

DNA methylation has been shown to be a marker of cancer [10] and occurs early in the development of breast cancer [11]. DNA methylation also plays an important role in regulating LNM. Clinical DNA methylation data can be used to identify biomarkers for predicting LNM of gastric cancer [12]. Identification and validation of DNA methylation markers could predict LNM in esophageal squamous cell carcinoma [13]. Epithelial-specific SHP1-P2 methylation can be used as a new universal tumor marker to detect LNM in colorectal cancer [14]. That promoter methylation downregulates RECK is associated with LNM in NSCLC [15]. Down-regulation of miR-148a by DNA methylation promotes metastasis and is associated with prognosis of skin cancer [16]. However, few studies have been conducted on methylation in ALNM of breast cancer and there are currently no molecular markers for evaluating ALNM in breast cancer.

In this study, we investigated genome-wide DNA methylation profiles of ALNM in breast cancer by RRBS. Compared with controls, patients with breast cancer with ALNM showed altered methylation levels in several genes. Promoter methylation of RASGRF2, AKR1B1, CRMP1 and RHOF in lymph node positive breast cancer tissues was significantly different from that in lymph node negative breast cancer tissues, which provides an important biological base for understanding lymph node positive breast cancer and developing therapeutic targets for lymph node positive breast cancer. Our results will advance knowledge and understanding of ALNM methylation groups in breast cancer.

## Materials and methods

### Patients and sample collection

The study was approved by the Ethics Institution Review Committee of Shenzhen Maternity $ Child Healthcare Hospital [SMCHEA (2017) No.13] and received written informed consent from all patients participating in the study. We continuously collected 19 breast cancer tissues with ALNM and 13 breast cancer tissues without ALNM from October 2017 to July 2020. We had access to information that could identify individual participants during and after data collection. The clinicopathological features of breast cancer were recorded by reviewing medical records (Table 1). All samples were from female patients with breast cancer. The mean ages of the LN positive group and the LN negative group were 44.5 and 44.1 years old, respectively. The P-value of the age between the two groups was 0.85 by t-test, indicating that there was no statistical difference in age between the two groups. All samples were collected during surgery for preliminary pathological examination and stored at -80°C for later use.

**Table 1:** Characteristics of patients with breast cancer.

Patient characteristics	LN positive breast cancer (*n* = 19)	LN negative breast cancer (*n* = 13)
Age (mean ± SD)	44.5±6.8	44.1±6.3
Nodes (N)	NO (0)	NO (13)
	N1–3 (19)	N1–3 (0)
Stage (T)	T1 (5)	T1 (4)
	T2 (14)	T2 (9)
Grading	Grade 2 (13)	Grade 2 (7)
	Grade 3 (6)	Grade 3 (6)
Estrogen receptor (ER)	ER– (2)	ER– (3)
	ER+ (17)	ER+ (10)
Progesterone receptor (PR)	PR– (2)	PR– (3)
	PR+ (17)	PR+ (10)
HER2 status	HER2– (15)	HER2– (11)
	HER2+ (4)	HER2+ (2)

LN, Lymph node; SD, Standard Deviation.

### Isolation and bisulfite modification of DNA

Genomic DNA was isolated from fresh frozen tissue samples by using TIANamp Micro DNA Kit (TIANGEN, Cat. No. DP316), according to the manufacturer’s instructions. Then genomic DNA was quantified using NanoDrop and Qubit Fluorometer, and the size was assessed by agarose gel electrophoresis.

### Reduced representation bisulfite sequencing

The Reduced Representation Bisulfite Sequencing (RRBS) procedure has been described previously [17]. Genomic DNA was subjected to RRBS library preparation using the Acegen Rapid RRBS Library Prep Kit (Acegen, Cat. No. AG0422) according to the manufacturer’s protocol. In brief, 100 ng of genomic DNA was digested with MspI, end-repaired, 3’-dA-tailed and ligated to 5-methylcytosine-modified adapters. After bisulfite treatment, the DNA was amplified with 12 cycles of PCR using Illumina 8-bp dual index primers. Size selection was performed to obtain DNA fractions of MspI-digested products in the range of 100–350 bp using a dual-SPRI® protocol according to the manufacturer’s protocol. The constructed RRBS libraries were then analyzed by Agilent 2100 Bioanalyzer and finally sequenced on Illumina Nova6000 platforms using a 150×2 paired-end sequencing protocol.

### Targeted bisulfite sequencing

Gene-specific DNA methylation was assessed by a next generation sequencing-based targeted bisulfite sequencing, according to previously published method [18–21]. In brief, primers were designed using the online MethPrimer software and listed in Table 2. 200 ng of genomic DNA was converted using the ZYMO EZ DNA Methylation-Gold Kit (Zymo Research, Irvine, CA, USA) and were used as templates for PCR amplification with 35 cycles using KAPA HiFi HotStart Uracil+ ReadyMix PCR Kit (Kapa Biosystems, Wilmington, MA, USA). For each sample, PCR products of multiple genes were pooled equally, 5’-phosphorylated, 3’-dA-tailed and ligated to barcoded adapter using T4 DNA ligase (NEB). Barcoded libraries from all samples were sequenced on Illumina Nova6000 platform using a 150×2 paired-end sequencing protocol.

**Table 2:** The primers for targeted bisulfite sequencing.

Gene	Forward Primer	Reverse Primer
BNIP3	TGCAAAYGYGGTGTGGGGYGGGCGTC	ACCTTAACACTTAAATTTAAATCAACAATACTTTC
PAX6	AGTTTGAGTTTTAAGGGGAGAGTTTA	CAACACCTTATCCATCTATTTTAAAAAC
GSTP1	GGAGTTYGYGGGATTTTTTAGAAGAG	TACTAAAAACTCTAAACCCCATCCCC
RHOF	GGGGTTAAAGTTTTAGTTGAATTTATTG	CATAATATACAACCAAAACTCCTTCC
PTOV1-AS1	GYGGGYGGATTATTTGAGGTTAGGAG	CTCATTTCCCAAACTAAAATACAATAATAC
ING5	AGTTTTYGAGTAGTTGGGAGTATAGG	CCTATAATCCCAACACTTTAAAAAACC
CRMP1	GTGGAGTTTAGGAGTGTTTTTTGAAT	TAATCCATTCATCCATCCACTCAATC
RASGRF2	GTATGTATTTTTTGGAGGGTTGTAGT	CTAATCCCCRAATTAAAACRACAAAAAAATAC
HOXA5	TTTTGTTTTGGGAGGYGGTTTGGGAG	CCTATACTAATATCTCTAAACTCCCC
AKR1B1	GGAGTTAAGGTTTTYGGTTTTTGTAAG	CATCCTAAAATTAAATACCTAAAAAATAAATACTC

### Data analyses

For RRBS experiments, sequencing adapters and low quality data of the sequencing data were removed by Trimmomatic [22]. The BSMAP software was used to map the bisulfite sequence to the reference genome [23]. The statistic information of the alignment was collected, only the unique mapped reads were kept for the following analysis, only methylated cytosines with sequence depth coverage of at least 5 were used. If the base on the alignment is C, methylation occurs; conversely, if the base on the alignment is T, no methylation occurs. The methylation levels of individual cytosines were calculated as the ratio of the sequenced depth of the ascertained methylated CpG cytosines to the total sequenced depth of individual CpG cytosines, i.e., ML = mC / (mC + umC). Where ML is the methylation level, mC and umC represent the number of reads supporting methylation C and the number of reads supporting unmethylated C, respectively. Differentially methylated regions (DMRs) analysis was performed using Metilene software [24]. DMR candidate regions were required to have average methylation level differences of >0.1 between the two groups. Finally, the regions with the 2D KS-test p-value <0.05 and BH (Benjamini & Hochberg) corrected p-value < 0.05 were determined as final DMRs. Here we use CG sites to look for differential methylation regions. The Gene Ontology (GO) project provides a controlled vocabulary to describe gene and gene product attributes in any organism (http://www.geneontology.org). The ontology covers 3 domains: biological process, cellular components, and molecular functions. Genes that exhibit at least 1 DMR were subjected to the GO enrichment analysis. Pathway analysis is a functional analysis mapping gene to the KEGG (Kyoto Encyclopedia of Genes and Genomes) pathway database (http://www.genome.jp/kegg/pathway.html).

For targeted bisulfite sequencing experiments, we use Trimmomatic 0.36 software to truncate the sequencing adapters and low-quality data of the sequencing data, and obtain clean data for subsequent analysis. Next, the clean data was aligned with the amplified target sequence, and the BSMAP 2.73 software was used for alignment. After the alignment was completed, the methylation level of the CG site was calculated using the python program for calculating methylation that comes with BSMAP. Finally, according to the methylation level calculated above, R language is used for statistical and visual display, and two-tailed t-test is used for inter group difference analysis. When p-value < 0.05 and the absolute value of the mean difference of methylation level between groups is greater than 0.1, the difference is considered significant.

## Results

### Differential methylation analysis of breast cancer tissues with and without ALNM

**Fig 1:**
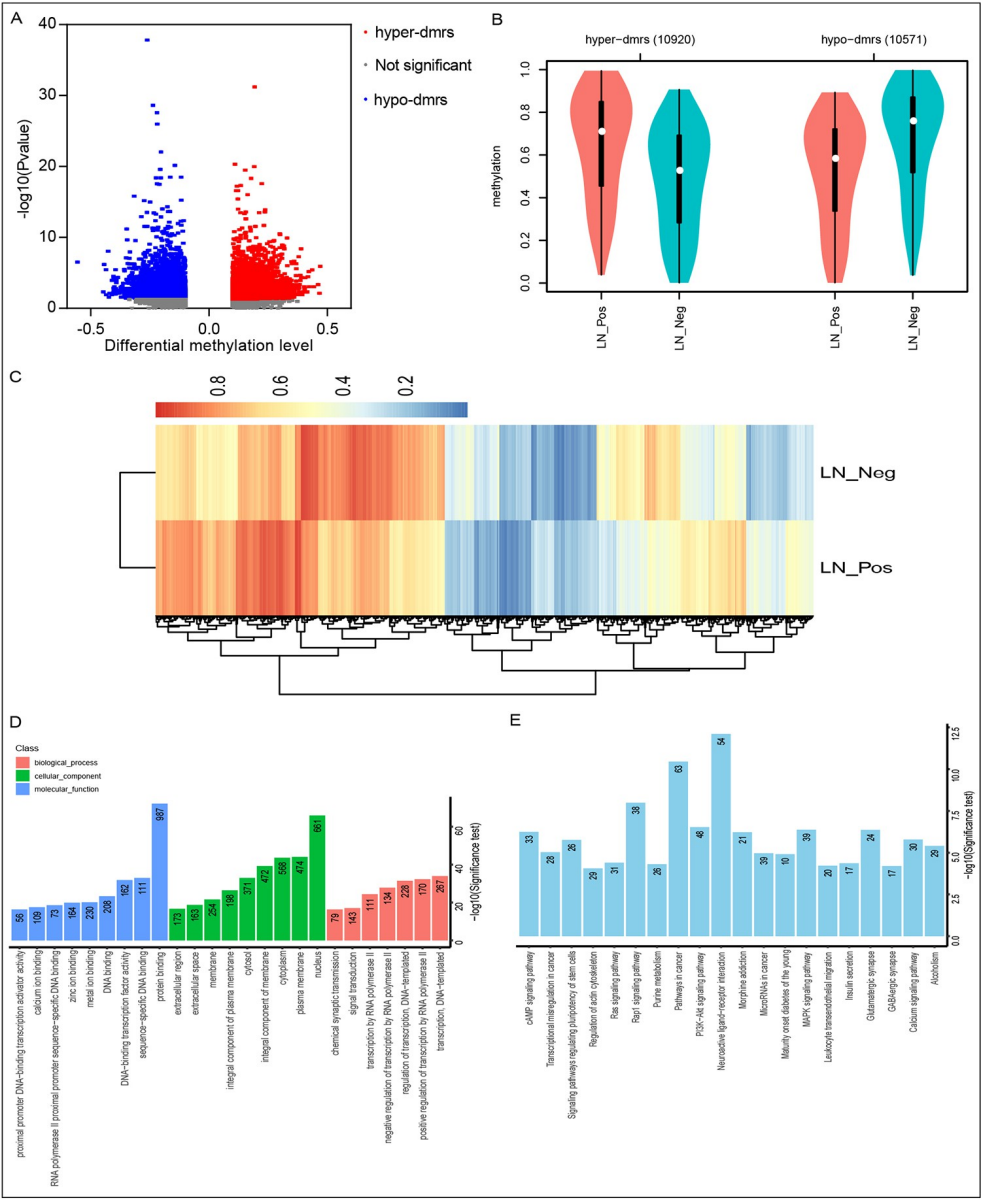
Differentially methylated regions (DMRs) analysis. A. Volcano plot showing mean methylation differences between LN positive and LN negative breast cancer (x axis) versus log transformed p-values (y axis). B. Violin boxplot showing the average methylation level distribution of DMRs. C. Cluster according to the average methylation level of the DMRs. D. The GO analysis of DMRs between LN positive and LN negative breast cancer. E. The KEGG analysis of DMRs between LN positive and LN negative breast cancer. LN, Lymph node; hyper-dmrs, hypermethylated DMRs; hypo-dmrs, hypomethylated DMRs.

We characterized DNA methylation patterns in breast cancer specimens, including breast cancers with and without ALNM. For DNA methylation analysis, we applied reduced representation bisulfite sequencing (RRBS), which can cover more than 6-8M CpG sites, >90% of CpG islands and >85% of gene promoters across the whole genome. We compared methylation differences between breast cancer tissues with and without ALNM, and identified 21491 differentially methylated regions (DMRs) between the two groups (S1 Table). Volcano plot and violin boxplot show that compared with the LN-negative breast cancer, LN-positive breast cancer has 10,920 hypermethylated DMRs and 10,571 hypomethylated DMRs (Fig 1A and 1B). A cluster heatmap of DMR methylation levels shows methylation status and differences between two groups (Fig 1C).

### Functional and signaling pathway analysis of differentially methylated genes

It is well known that methylation of the gene promoter region affects gene expression. Hypermethylation of the gene promoter region leads to gene inactivation, while hypomethylation of the gene promoter region leads to increased gene expression. Therefore, based on the distribution of DMR in the genome, the genes that overlapped in DMR and the gene promoter region (1.5K upstream of transcription start site to 0.5K downstream of transcription start site) were analyzed for functional enrichment analysis. According to Gene Ontology (GO) analysis, the differential methylation genes are involved in a variety of biological processes. We filter out the GO items with ovserved > 2, foldchange > 2 and P < 0.05, the top 25 GO terms that were most significantly enriched are shown in Fig 1D. KEGG (Kyoto Encyclopedia of Genes and Genomes) analysis shows that the differential methylation genes are involved in a variety of KEGG pathway. We filter out the KEGG pathway items with ovserved > 2, foldchange > 2 and P < 0.05, the top 20 KEGG pathway terms that were most significantly enriched are shown in Fig 1E. The GO and KEGG analysis showed that differential promoter methylation genes were mainly related to regulation of transcription and cancer-related pathways, such as MAPK signaling Pathway, PI3K-Akt signaling Pathway, and transcriptional misregulation in cancer.

### Aberration of CpG methylation on the promoter regions of RHOF, RASGRF2, AKR1B1, and CRMP1 genes as methylation biomarkers for lymphatic metastasis of breast cancer

**Fig 2:**
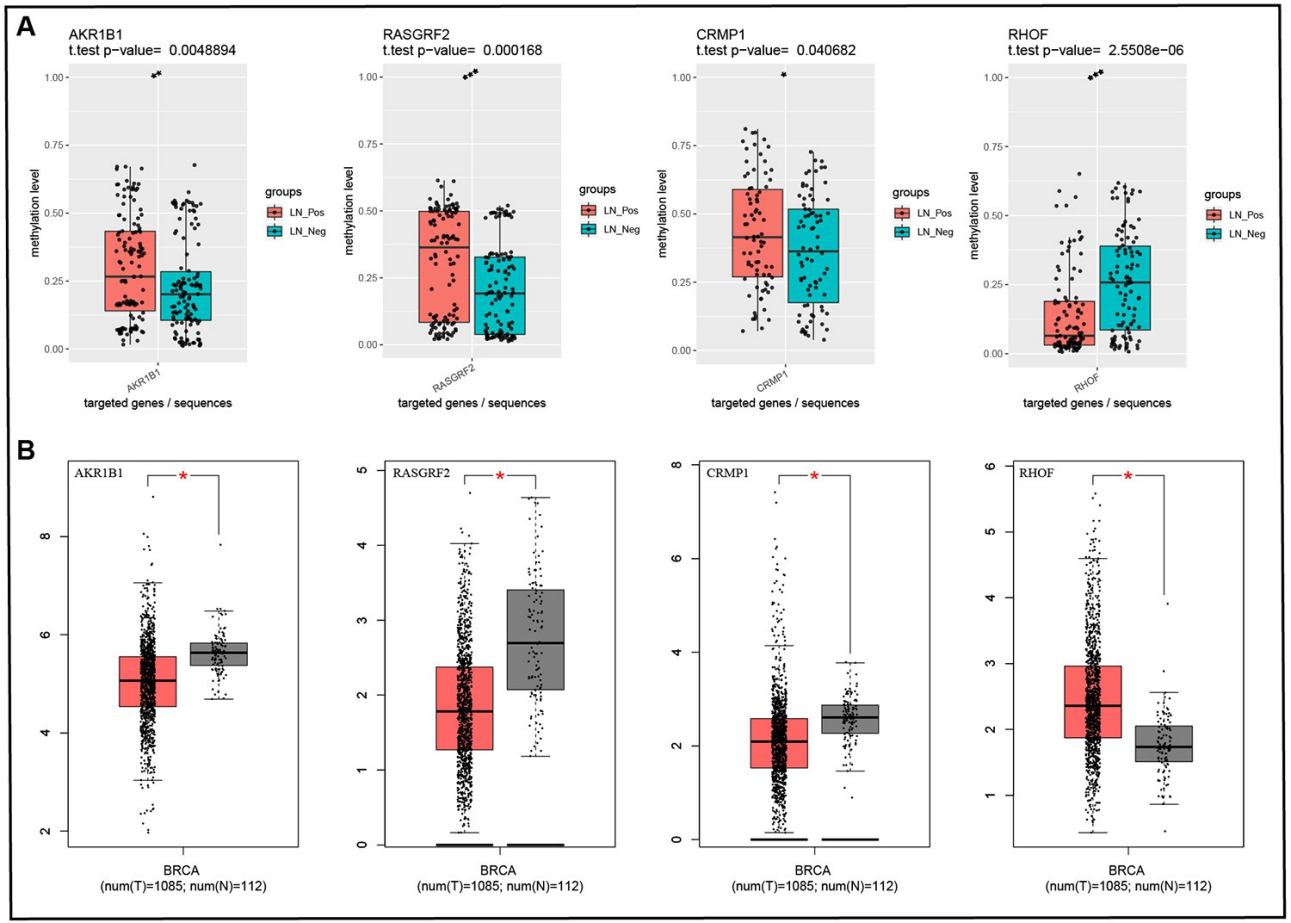
Box plots. A. Methylation difference between LN positive and LN negative breast cancer. B. Gene expression difference between breast cancer and normal. LN, Lymph node. T, Tumor. N, normal.

Based on the RRBS, we selected 10 DMRs in the promoter region for validation. These 10 DMRs covered 10 genes, including HOXA5, PTOV1-AS1, RHOF, PAX6, GSTP1, RASGRF2, AKR1B1, BNIP3, CRMP1, ING5. The DMRs identified from gene promoter regions by RRBS was validated by using targeted bisulfite sequencing in 12 clinical specimens. Primer sequences and product sizes are shown in Table 2. Methylation levels of each site in each sample were obtained by targeted bisulfite sequencing, as shown in S2 Table. And the average methylation level of each amplicon was calculated (Table 3). Targeted bisulfite sequencing showed that the promoter methylation levels of RASGRF2, AKR1B1 and CRMP1 were increased in positive lymph node compared with negative lymph node breast cancer tissues, while the promoter methylation level of RHOF weas decreased in positive lymph node breast cancer tissues (Fig 2A). In addition, we analyzed the expression of these 4 genes in breast cancer tissues using data from Cancer Genome Atlas (TCGA) and draw box plots using GEPIA (http://gepia.cancer-pku.cn/index.html) [25], which showed that RASGRF2, AKR1B1 and CRMP1 were low expressed in breast Cancer tissues, while RHOF was high expressed in breast Cancer tissues (Fig 2B).

**Table 3:** List of 10 amplicon in the promoter region between LN positive and LN negative breast cancer by using targeted bisulfite sequencing.

Gene	Mean (LN positive)	Mean (LN negative)	Diff	p.value
AKR1B1	0.295357	0.230302	0.065056	0.004889
BNIP3	0.422614	0.445813	-0.0232	0.668368
CRMP1	0.430077	0.36309	0.066987	0.040682
GSTP1	0.091754	0.091889	-0.00013	0.99286
HOXA5	0.663654	0.629641	0.034013	0.250466
ING5	0.228241	0.228389	-0.00015	0.994112
PAX6	0.4285	0.385487	0.043013	0.12395
PTOV1-AS1	0.532967	0.517853	0.015114	0.742656
RASGRF2	0.301219	0.208202	0.093018	0.000168
RHOF	0.142392	0.257941	-0.11555	2.55E-06

p.value < 0.05 indicates significantly differences between the two groups.

Mean, average methylation level; Diff, differential methylation levels; DMRs, differentially methylated regions; LN, Lymph node.

### Aberration of CpG methylation on the promoter regions of AKR1B1, BNIP3, CRMP1, GSTP1, HOXA5, PAX6 and RASGRF2 genes is associated with ALNM in ER-positive and HER2-negative breast cancer

**Fig 3:**
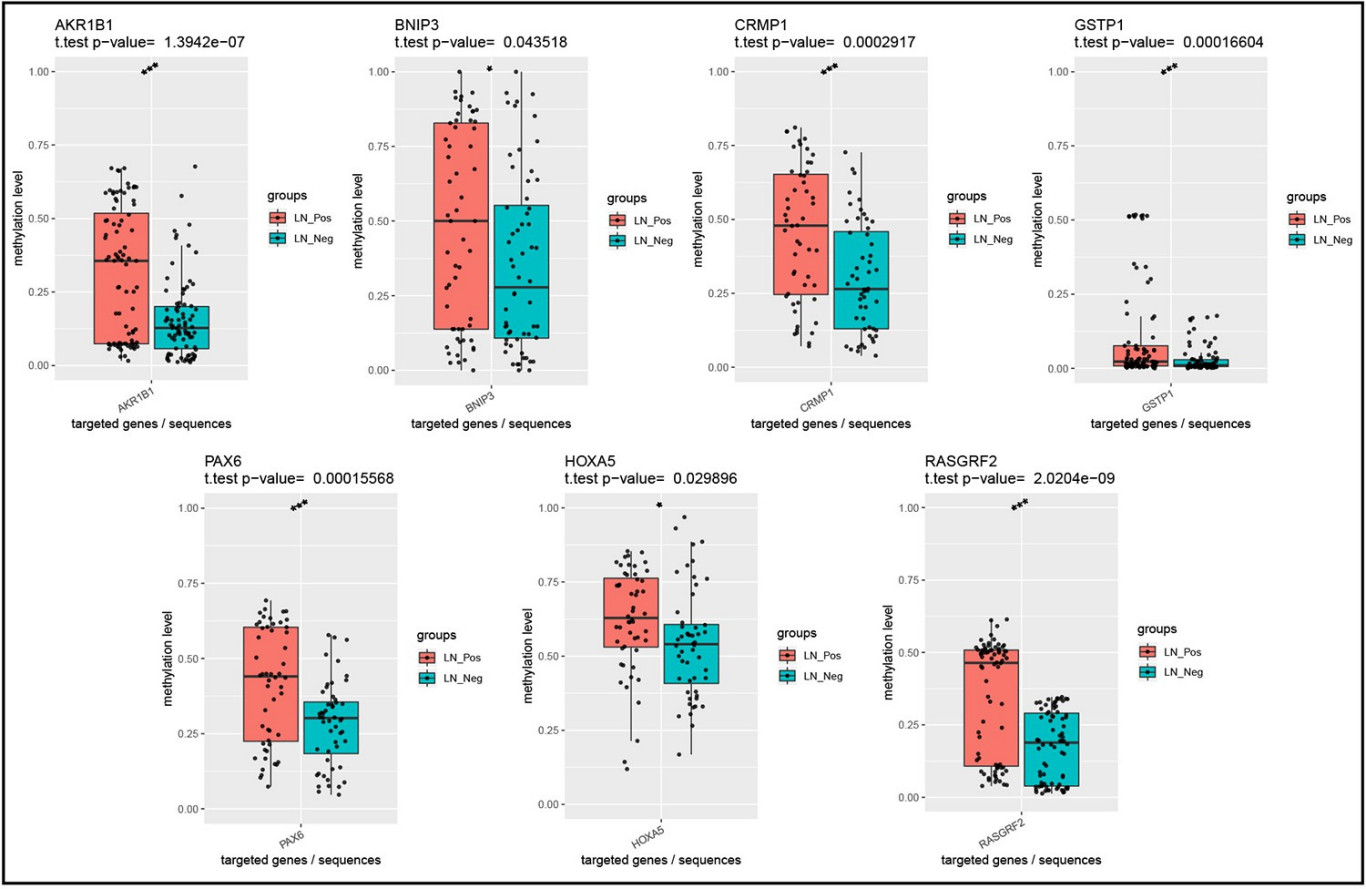
Box plot show differences between ER-positive and HER2-negative breast cancers with and without axillary lymph node metastasis. LN, Lymph node.

Estrogen receptor (ER) positive breast cancer has a relatively good prognosis, while ER negative breast cancer has a relatively poor prognosis. The relative prognosis of breast cancer is relatively poor when human epidermal growth factor receptor-2 (HER2) is positive, and relatively good when HER2 is negative. Therefore, we compared methylation levels of the 10 gene promoters in 8 ER-positive and HER2-negative breast cancers with or without ALNM from the 12 clinical samples mentioned above. These genes included HOXA5, PTOV1-AS1, RHOF, PAX6, GSTP1, RASGRF2, AKR1B1, BNIP3, CRMP1, ING5. Methylation levels of each site in each sample were obtained by targeted bisulfite sequencing, as shown in S3 Table. And the average methylation level of each amplicon was calculated (Table 4). Targeted bisulfite sequencing showed that the promoter methylation levels of AKR1B1, BNIP3, CRMP1, GSTP1, HOXA5, PAX6, and RASGRF2 were increased in ER-positive and HER2-negative breast cancers with ALNM, compared with ER-positive and HER2-negative breast cancers without ALNM (Fig 3).

**Table 4:** List of 10 amplicon in the promoter region between ER-positive and HER2-negative breast cancers with and without axillary lymph node metastasis by using targeted bisulfite sequencing.

Gene	Mean (LN positive)	Mean (LN negative)	Diff	p.value
AKR1B1	0.313321	0.157143	0.156179	1.39E-07
BNIP3	0.477088	0.356967	0.120121	0.043518
CRMP1	0.454962	0.300788	0.154173	0.000292
GSTP1	0.102917	0.03019	0.072726	0.000166
HOXA5	0.619769	0.541827	0.077942	0.029896
ING5	0.228556	0.230444	-0.00189	0.939425
PAX6	0.413385	0.283731	0.129654	0.000156
PTOV1-AS1	0.51835	0.528292	-0.00994	0.847474
RASGRF2	0.346711	0.174145	0.172566	2.02E-09
RHOF	0.152338	0.175588	-0.02325	0.403496

p.value < 0.05 indicates significantly differences between the two groups.

Mean, average methylation level; Diff, differential methylation levels; DMRs, differentially methylated regions; LN, Lymph node.

## Discussion

DNA methylation is one of the most important epigenetic mechanisms and plays an important role in regulating the cancer metastasis. To investigate the role of DNA methylation in ALNM of breast cancer, we conducted the RRBS experiment. RRBS is a cost-effective method for generating genome-wide DNA methylation maps at single-nucleotide resolution [26]. We used RRBS analysis to find that lymphatic metastasis and non-lymphatic metastasis had the same methylation distribution in breast cancer. The lymphatic metastasis and the non-lymphatic metastasis have different levels of methylation. Many DMRs were detected in two experimental groups. Compared to the LN-negative breast cancer, LN-positive breast cancer had 10,920 hypermethylated DMRs and 10,571 hypomethylated DMRs. We assessed the differential methylation genes by GO and KEGG analysis and found that the differential methylation genes were mainly related to regulation of transcription and cancer-related pathways, such as MAPK signaling Pathway, PI3K-Akt signaling Pathway, and transcriptional misregulation in cancer. Therefore, we concluded that genes with abnormal promoter methylation in lymph node breast cancer could affect transcriptional regulation and be related to the occurrence of cancer.

Next, we carried out targeted bisulfite sequencing experiment to verify several DMRs, including HOXA5, PTOV1-AS1, RHOF, PAX6, GSTP1, RASGRF2, AKR1B1, BNIP3, CRMP1, ING5. The results showed that compared with negative lymph node breast cancer tissues, RHOF promoter methylation levels were decreased in positive lymph node breast cancer tissues, while AKR1B1, RASGRF2, and CRMP1 gene promoter methylation levels were increased. But TCGA data showed that RASGRF2, AKR1B1 and CRMP1 were low expressed in breast Cancer tissues, while RHOF was high expressed in breast Cancer tissues. And compared with ER-positive and HER2-negative breast cancers without ALNM, the methylation levels of AKR1B1, RASGRF2, CRMP1, BNIP3, GSTP1, HOXA5, and PAX6 promoter were increased in ER-positive and HER2-negative breast cancers with ALNM.

Some studies have found that the methylation of GSTP1, AKR1B1, RASGRF2 and PAX6 promoter play an important role in tumorigenesis. Previous articles showed that hypermethylation of GSTP1 promoter was significantly related to LNM. GSTP1 is a tumor suppressor gene, hypermethylation of GSTP1 was significantly associated with patients having macroscopic sentinel LNM compared with those with microscopic or no sentinel node metastasis in breast cancer [27], which is consistent with our results. Studies have found that the promoters of the AKR1B1 and RASGRF2 genes were highly methylated in breast cancer, which may serve as candidate methylation biomarkers for early breast cancer detection [28, 29]. The study showed methylation of PAX6 was closely related to LNM in breast cancer, esophageal squamous cell carcinoma and gastric cancer [13, 30, 31]. However, methylation of RHOF, CRMP1, BNIP3 and HOXA5 promoters has not been reported to be associated with lymph node metastasis, and our study is the first to find that methylation of these gene promoters is associated with lymph node metastasis. But many studies have shown that the expression of these genes is related to lymph node metastasis. The RHOF expression was associated with tumor-lymph node-metastasis stage, T grade, metastatic status, recurrence, and survival of hepatocellular carcinoma, and RHOF plays a key role in promoting HCC cell migration, invasion and EMT by regulating Warburg effect [32]. CRMP1 is a tumor suppressor, and low expression of CRMP1 mRNA in lung cancer tissue was significantly associated with disease progression, LNM, early postoperative recurrence, and shorter survival [33]. BNIP3 is a tumor suppressor, the expression of BNIP3 was significantly reduced in pancreatic cancer and is associated with tumor size, clinical stage and LNM [34]. Overexpression of HOXA5 inhibited cell proliferation and invasion, and its expression level was significantly correlated with tumor-node-metastasis stages, tumor size, and LNM in non-small-cell lung carcinoma [35].

Hypermethylation of promoter leads to gene inactivation, while hypomethylation leads to increased gene expression. It is possible to regulate lymph node metastasis of breast cancer by affecting these genes closely related to lymph node metastasis through promoter methylation. Our study found hypomethylation of the RHOF promoter in lymph node metastatic breast cancer tissue, which is likely to lead to elevated RHOF expression in positive lymph node tissue compared to negative lymph node tissue. However, elevated RHOF expression is likely to lead to ALNM of breast cancer. Therefore, methylation of RHOF promoter has potential as a therapeutic target and prognostic biomarker for breast cancer with ALNM. AKR1B1 and RASGRF2 were highly methylated in metastatic breast cancer [28], and our study showed that promoters of AKR1B1 and RASGRF2 were also hypermethylated in breast cancer with ALNM, which suggested that AKR1B1 and RASGRF2 may play the same role in ALNM breast cancer as in metastatic breast cancer and may also be candidate methylation biomarkers for breast cancer with ALNM. Our study also found that the CRMP1 promoter is highly methylated in lymph node metastatic breast cancer tissues, which may result in lower CRMP1 expression in positive lymph node tissues than in negative lymph node tissues. However, low expression of CRMP1 may lead to ALNM of breast cancer. Therefore, methylation of CRMP1 promoter may serve as a therapeutic target and prognostic biomarker for breast cancer with ALNM. Furthermore, in addition to highly methylated AKR1B1, RASGRF2 and CRMP1 gene promoters, BNIP3, GSTP1, HOXA5 and PAX6 gene promoters were also methylated in ER-positive and HER2-negative breast cancer with ALNM, which may serve as candidate methylation biomarkers. Considering the small number of ER-positive and HER2-negative breast cancer samples, more studies with larger sample size are needed to further confirm the relationship between methylation of these genes and lymph node metastasis.

## Conclusion

In conclusion, we found that there were different methylation states between breast cancer tissues with and without ALNM by RRBS. Targeted bisulfite sequencing showed that promoter methylation of RASGRF2, AKR1B1, CRMP1 and RHOF in lymph node positive breast cancer tissues was significantly different from that in lymph node negative breast cancer tissues. Moreover, in addition to highly methylated AKR1B1, RASGRF2 and CRMP1 gene promoters, BNIP3, GSTP1, HOXA5 and PAX6 gene promoters were also methylated in ER-positive and HER2-negative breast cancer with ALNM. Methylation of PAX6, GSTP1, RASGRF2, and AKR1B1 promoters has been previously reported to be associated with lymph node metastasis of breast cancer. However, our study found for the first time that methylation of RHOF, CRMP1, BNIP3 and HOXA5 promoters is associated with lymph node metastasis. Therefore, our study identified RHOF, CRMP1, BNIP3 and HOXA5 as novel candidate methylation biomarkers, which can be used to predict ALNM in breast cancer or ER-positive and HER2-negative breast cancer. The next step will be to assess the methylation levels of these genes in distant metastasis of breast cancer.

## Supporting information

S1 Table Differentially methylated regions between breast cancer tissues with and without axillary lymph node metastasis. (XLS)

S2 Table Methylation levels at each site in each breast cancer sample. (XLS)

S3 Table Methylation levels at each site in each ER-positive and HER2-negative breast cancer sample. (XLS)

## List of abbreviations

ALNM: axillary lymph node metastasis
DMRs: differentially methylated regions
RRBS: reduced representation bisulfite sequencing
LN: lymph node
